# Research Progress on the Treatment of Premature Ovarian Failure Using Mesenchymal Stem Cells: A Literature Review

**DOI:** 10.3389/fcell.2021.749822

**Published:** 2021-12-13

**Authors:** Jing Wang, Wanru Liu, Dehai Yu, Zongxing Yang, Sijie Li, Xiguang Sun

**Affiliations:** ^1^ Department of Reproductive Medicine, Department of Prenatal Diagnosis, The First Hospital of Jilin University, Changchun, China; ^2^ The Laboratory of Cancer Precision Medicine, The First Hospital of Jilin University, Changchun, China; ^3^ Department of Clinical Laboratory, The First Hospital of Jilin University, Changchun, China; ^4^ Department of Breast Surgery, The First Hospital of Jilin University, Changchun, China; ^5^ Hand Surgery Department, The First Hospital of Jilin University, Changchun, China

**Keywords:** mesenchymal stem cells, fertility, premature ovarian failure (POF), ovarian dysfunction, reproductive medicine

## Abstract

Premature ovarian failure (POF) has become one of the main causes of infertility in women of childbearing age and the incidence of POF is increasing year by year, seriously affecting the physical and mental health of patients and increasing the economic burden on families and society as a whole. The etiology and pathogenesis of POF are complex and not very clear at present. Currently, hormone replacement therapy is mainly used to improve the symptoms of low estrogen, but cannot fundamentally solve the fertility problem. In recent years, stem cell (SC) transplantation has become one of the research hotspots in the treatment of POF. The results from animal experiments bring hope for the recovery of ovarian function and fertility in patients with POF. In this article, we searched the published literature between 2000 and 2020 from the PubMed database (https://pubmed.ncbi.nlm.nih.gov), and summarized the preclinical research data and possible therapeutic mechanism of mesenchymal stem cells (MSCs) in the treatment of POF. Our aim is to provide useful information for understanding POF and reference for follow-up research and treatment of POF.

## Introduction

POF is a kind of ovarian dysfunction characterized by menstrual disorder, ovarian atrophy, decreased sexual life and decreased fertility in women between puberty and 40 years old, which seriously affects female reproductive health and endocrine balance and is one of the main causes of female infertility (Sheikhansari et al., 2018). Approximately 1% of women under the age of 40 suffer from premature ovarian failure (Huhtaniemi et al., 2018). Under the influence of high pressure and a fast paced life, the incidence of POF is increasing and manifesting at younger ages, and it has affected more than 10% of women in recent years (Thakur et al., 2018).

POF treatment is extremely difficult. Although assisted reproductive technology has become an effective treatment, it is not ideal, and fertility loss and low estrogen status have become a great threat to female reproductive health (Laven, 2016). POF has become one of the most severe problems threatening the reproductive health of women of normal childbearing age. Its occurrence may be related to an insufficient reserve of primordial follicular cistern, accelerated follicular atresia, changes of dominant follicular recruitment, follicular maturation disorders and so on (Xiang et al., 2019). In view of the limitations of conventional treatment, clinical and scientific research work has focused on improving ovarian function and restoring fertility in patients with POF. In recent years, MSC transplantation has opened up a new direction for the treatment of POF, but this is still in the stage of preclinical research (Lai et al., 2015; Sun et al., 2017; Zhang et al., 2018; Liu S. et al., 2019; Zheng et al., 2019), and there are few clinical studies so far. The mechanism by which MSCs improve ovarian function has also not been completely elucidated. At present, there is no clear and effective treatment to restore the reproductive function of ovaries. In this paper, we reviewed the preclinical research data of the treatment of POF using MSCs and the possible therapeutic mechanisms to provide a reference for follow-up research and treatment of POF.

## The Current Situation of POF Treatment

**FIGURE 1:**
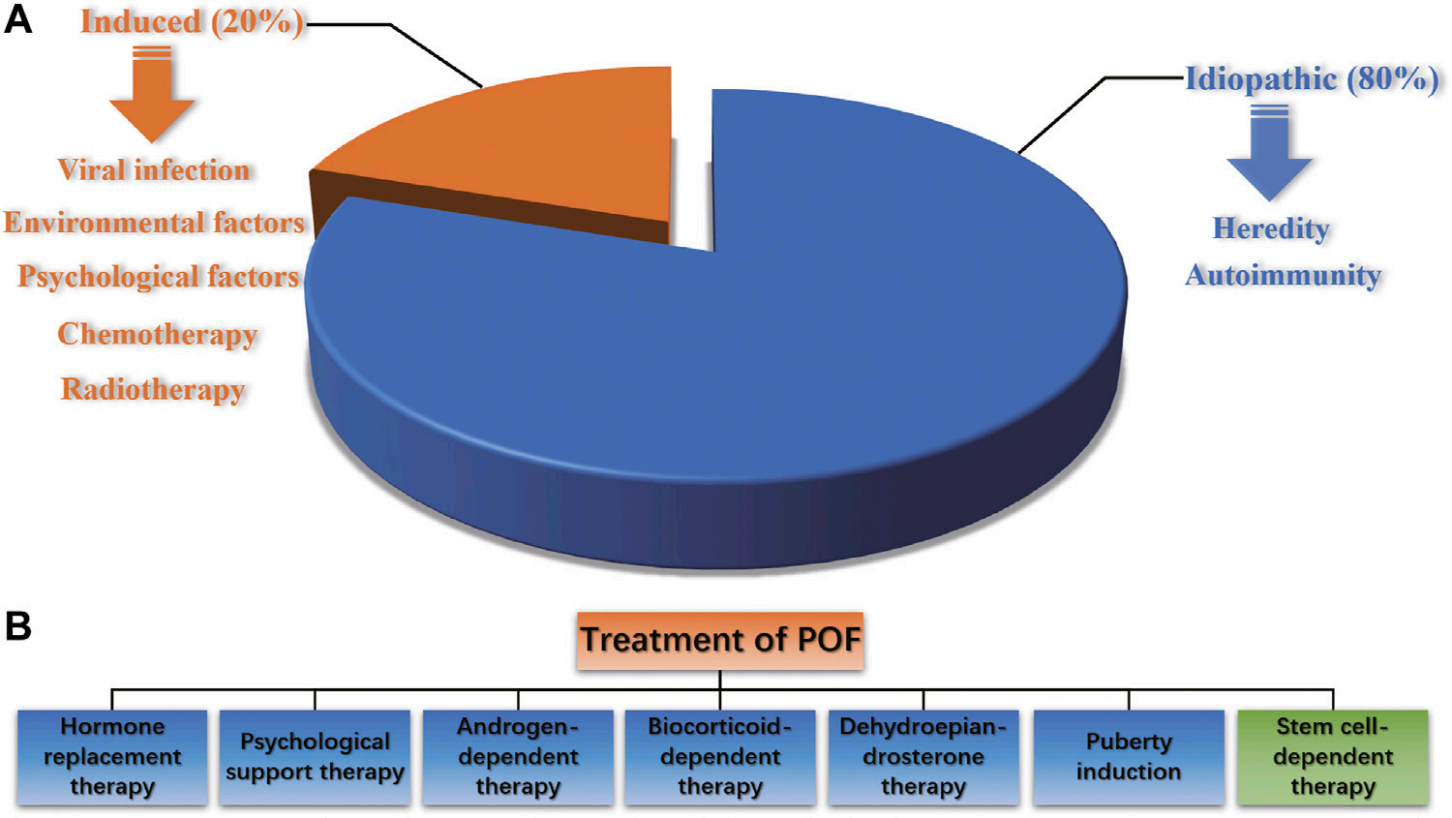
The pathogenic factors and treatment options of POF.

POF is a reproductive endocrine disease that occurs before the age of 40 and is characterized by increased gonadotropin levels and decreased estrogen levels, accompanied by primary or secondary amenorrhea. It is also one of the common diseases leading to female infertility. POF is a highly heterogeneous condition. Abnormal follicular development in all stages can lead to POF, and such damage to ovarian function is irreversible. The pathogenic factors of POF include heredity, autoimmunity, viral infection, iatrogenic factors, and environmental and psychological factors, and approximately eighty percent of POF cases are idiopathic (Webber et al., 2016) (Figure 1A). It has been reported that radiotherapy, chemotherapy and bone marrow transplantation of cancer can result in POF (Dolmans and Donnez, 2021; Imai et al., 2008). The traditional treatment of POF includes hormone replacement therapy (HRT), psychological support therapy, androgen-dependent therapy, biocorticoid-dependent therapy, dehydroepiandrosterone therapy and puberty induction (Figure 1B). However, HRT can only relieve low estrogen symptoms such as vaginal dryness, hot flashes and genitourinary tract atrophy, but has no essential effect on improving ovarian reproductive function. Long-term use of HRT is controversial because it increases the risk of endometrial and ovarian cancer (Ali, 2013; Lee et al., 2020). Since the etiology of POF infertility is complex, the current treatment efficacy is unsatisfactory, and the pregnancy rate and carrying to term rates are still quite low after treatments. Therefore, for women with fertility requirements, it is necessary to strengthen early prevention, early detection and early treatment to delay the development of POF and improve the live birth rate.

The results from animal experiments of MSC transplantation has brought hope to the recovery of ovarian function and fertility in patients with POF. In the following, we will introduce advances in the treatment of POF with MSCs.

## MSCs and Fertility Protection

MSCs were the first type of adult stem cell discovered in bone marrow. They originate from mesoderm and are distributed in almost all connective tissue and organ stroma of the entire body. They have the potential for multidirectional differentiation of stem cells and also have a strong migration ability to damaged tissues. Since MSCs have low immunogenicity and fewer disputes in bioethics than fetal-derived stem cells, they are widely applied in clinical research and medical bioengineering (Pers et al., 2016; Badawy et al., 2017; Mu et al., 2018). Currently, MSCs have been used to treat diseases related to the blood, nervous, motor, cardiovascular and skin systems, showing good curative effects (Zaher et al., 2014).

The reproductive capacity of most female mammals is mainly affected by the primordial follicular pool. Under normal circumstances, to avoid depletion of the follicular pool, most primordial follicles in the ovary are maintained in a resting phase. Primordial follicles undergo follicular activation and a series of developmental processes and finally develop into mature follicles. Various molecules are involved in regulating follicular activation, growth and atresia. Ovarian function recovery is based on oocyte production and follicular quantity/quality recovery (Woods and Tilly, 2012; Truman et al., 2017). Several studies have shown that MSCs can directly differentiate into oocyte-like cells, and transplantation of MSCs is conducive to restoring ovarian function and reproductive capacity (Bahrehbar et al., 2020; Yoon et al., 2020; Taheri et al., 2021). Therefore, MSCs are considered a new choice for the treatment of POF.

The effectiveness of MSCs in the treatment of reproductive system diseases has been confirmed by preclinical and clinical research, which has brought great hope to POF infertility and improved female reproductive health (Herraiz et al., 2019; Fu et al., 2021; Li et al., 2021). MSCs used for the treatment of POF include BMSCs, UCMSCs, PMSCs, AMSCs, AFMSCs, MenSCs and ADMSCs. MSCs originating from different sources have some common characteristics, which make them an ideal treatment choice for POF. A number of animal experiments and clinical trials have confirmed that ovarian function can be improved by MSC homing, inhibiting the apoptosis of OGC and promoting ovarian angiogenesis (Esfandyari et al., 2020). For example, Yan et al. transplanted MSCs to 61 patients with POF and found that the number of follicles in each developmental stage, including antral follicles, dominant follicles and mature follicles, increased significantly (Yan et al., 2020). Other researchers have found that autologous MSC transplantation can trigger menstruation to resume, relieve menopausal symptoms, improve ovarian function and help patients become pregnant (Bukovsky and Caudle, 2012; Igboeli et al., 2020; Mashayekhi et al., 2021; Ulin et al., 2021). Ling *et al.* treated POF mice with MSCs and found that MSC transplantation could significantly restore their hormone secretion ability, improve follicular growth and GC survival, and recover the ovarian function that was destroyed by chemotherapy used to create the POF mice (Ling et al., 2019). A meta-analysis of POF indicated that MSCs could decrease the level of FSH, increase the level of E2 and promote the proliferation of follicles, thus improving the quality of ovaries in POF animals and humans (Chen et al., 2018). Interestingly, Bahrehbar et al*.* proved that MSC-transplanted POF mice can produce offspring (Bahrehbar et al., 2020).

Table 1 summarizes the preclinical and clinical trials that indicate the validity of treating POF with MSCs. However, the underlying molecular and cellular mechanisms are still controversial and need to be further clarified. Additionally, current clinical research is still insufficient, and there is still a long way to go before the large-scale clinical application of MSCs.

**TABLE 1:** Advances in the treatment of POF with MSCs.

Research category	Type of MSCs	Method	Outcome of MSC treatment	Molecular mechanism	Biological effect	References
Preclinical research/animal experiment	Mouse menSCs	Injection by the tail vein	Repairing ovarian injury, improving ovarian function and stimulating regeneration	MenSCs produce high level of FGF2, which is essential for angiogenesis and the proliferation and remodeling of endometrial cells that plays important roles in repairing and regenerating the damaged tissues	MenSCs increase the follicular numbers, return sex hormone level, repair oocyte function and protect ovary damage	Wang et al. (2017)
Preclinical research/animal experiment	Human PMSCs	Injected subcutaneously	Restoring ovarian function	PMSCs activate the PI3K/Akt pathway, reduce Th17 cells percentage and increase Treg cells percentage	PMSCs increase serum levels of E2 and AMH and decrease FSH, LH and AZPAb levels	Yin et al. (2018b)
Preclinical research/animal experiment	Human AMSCs	Intraperitoneal injection and intragastric administration	Improving injured ovarian tissue structure and function	AMSC transplantation elevate serum oestrogen level and decrease FSH secretions	AMSCs promote follicular development, granulosa cell proliferation and secretion function by improving the local microenvironment of POF mouse ovary	Liu et al. (2019b)
Preclinical research/animal experiment	Mouse ADSCs	Intravenous injection	Improving ovarian function	Expression levels of ZCCHC11, ANGPTL and ONECUT2 are upregulated	ADSCs increase follicle number, ovulation and inhibit cell apoptosis in POF ovaries	Sun et al. (2013)
Preclinical research/laboratory research	Human BMSCs	Collection of MSC conditioned media	—	BMSCs conditioned media increase angiogenesis marker including VEGF, VEGFR, Endoglin, Tie-2 and VE-Cadherin through the PI3K/ALK pathway	MSC conditioned media stimulates the proliferation of HOVEC cells	Park et al. (2019)
Preclinical research/animal experiment	Human BMSCs	Intraovarian injection	Restoring ovarian hormone production and reactivating folliculogenesis	BMSCs decrease FSH level and increase AMH level	BMSCs induce follicle growth and increase the pregnancy rate	Mohamed et al. (2018)
Preclinical research/animal experiment	Human UCMSC	Intraovarian injection	UCMSC transplantation preserved ovarian function of POF mice	UCMSC transplantation increase estrogen (E2) and AMH levels, and increase the expression of CD31	UCMSCs increase ovarian volume and the number of antral follicles, and promote granulosa cell proliferation and ovarian angiogenesis	Yang et al. (2019b)
Clinical research	Human UCMSC	Intraovarian injection	Two POF patients conceived naturally within 1 year after UCMSC transplantation	UCMSCs activate primordial follicles via phosphorylation of FOXO3a and FOXO1	UCMSCs rescue ovarian function, elevate estradiol concentrations, improve follicular development and increase the number of antral follicles	Ding et al. (2018)
Clinical research	Human autologousBMSC	Laparoscopic intraovarian injection	BMSC treatment revealed promising improvement of POF.	—	BMSCs elevate serum estrogen level, increase volume of the treated ovaries and improve menopausal symptoms	Igboeli et al. (2020)
Clinical research	Human autologous BMSC	Intraovarian instillation	Perimenopausal woman delivered a healthy baby	BMSCs increase AMH level	BMSCs improve follicular development	Gupta et al. (2018)
Clinical research	Human autologous BMSC	Intraarterial catheterization to ovarian artery	5/15 poor responders conceived and 3 healthy babies were born after the stem cell administration	BMSCs increase AMH level and antral follicular count	BMSCs increase the number of antral follicles and retrieve oocytes	Herraiz et al. (2018)
Clinical research	Human autologous ADSCs	Intraovarian injection	Menstruation resumption	BMSCs decreased FSH level	—	Mashayekhi et al. (2021)
Clinical research	Human UCMSC	Intraovarian injection	UCMSC transplantation improved the injured ovarian function, and 4/61 POI patients obtained clinical delivery	—	UCMSCs increase follicular development and improve egg collection	Yan, et al. (2020)

## The Mechanism of Treating POF With MSCs

**FIGURE 2:**
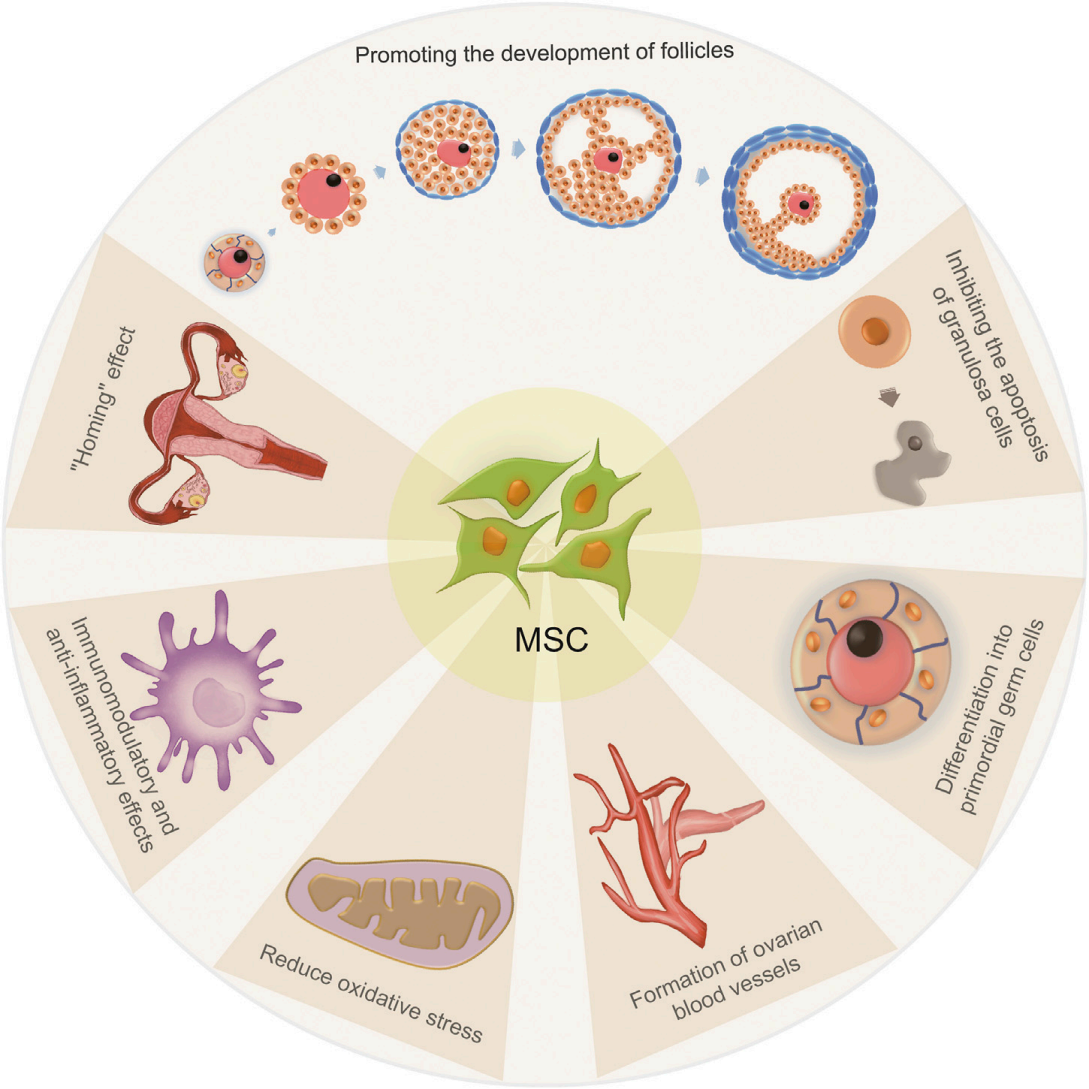
The mechanisms of treating POF with MSCs.

The mechanism of treating POF with MSCs can be summarized as follows (Figure 2): 1) MSCs have a “homing” effect; 2) MSCs can promote the growth and development of follicles at all developmental stages; 3) MSCs may induce and differentiate into primordial germ cells (uncertain); 4) MSCs can directly differentiate into GCs or inhibit the apoptosis of GCs; 5) MSCs can promote the formation of ovarian blood vessels; 6) MSCs have immunomodulatory and anti-inflammatory effects and 7) MSCs can reduce oxidative stress.

### Homing Effect of MSCs

The homing capacity of MSCs is an important determinant of effective MSC-based therapy (Li et al., 2017; Lin et al., 2017). Homing refers to the process by which MSCs migrate to damaged tissues and promote their recovery. Therefore, enhancing the homing efficiency of MSCs is essential for optimizing the therapeutic outcome of POF. Noory *et al.* reported the application of MenSC transplantation as a treatment modality in a rat model of POF and observed that MSCs can survive in ovarian stroma at 2 months after MSC transplantation and directly differentiated into GCs (Noory et al., 2019). Experiments from Liu et al*.*, Jalalie, et al*.*, Lai et al., Song et al. and Park et al*.* also demonstrated that after transplantation, MSCs home to damaged tissue and reach the site of injured ovaries (Liu et al., 2014; Lai et al., 2015; Jalalie et al., 2019; Park et al., 2021). However, studies have also shown that although MSCs have a homing effect, they cannot directly differentiate into oocytes but do localize in the ovarian matrix, secrete various cytokines and improve ovarian reserve function through the paracrine pathway (Takehara et al., 2013; Gabr et al., 2016; Li et al., 2017). A study by Taheri et al*.* demonstrated that MSC isolated from follicular fluid cultured in human recombinant BMP15 medium may differentiate into oocyte-like cells *in vitro*, but they did not investigate whether such MSCs can differentiate into oocytes *in vivo* (Taheri et al., 2021). Therefore, whether MSCs can directly differentiate into oocytes remains unclear, and more in-depth laboratory experiments are still necessary to solve this scientific problem.

### Effects of MSCs on Follicular Development

Folliculogenesis is an important part of ovarian function, as it provides oocytes for reproduction (Hua et al., 2015). A large number of genes/proteins have been identified to be associated with follicular development, growth, ovulation and atresia processes. It has been reported that PMSC transplantation can increase the secretion of growth factors, angiogenic factors, pleiotropic cytokines, chemotactic cytokines and extracellular matrix proteins, which are all essential for folliculogenesis (Kupcova Skalnikova, 2013). In POF treatment, the widely discussed follicular development related genes are *Nanos3*, *Nobox* and *Lhx8*. Lai et al. proved that SMSC transplantation could reactivate injured mouse ovaries, with increased expression of the folliculogenesis marker genes *Nobox*, *Nanos3*, and *Lhx8* in the ovaries of SMSC-treated mice (Lai et al., 2014). Kim et al. showed that three-dimensional cultured PDMSC spheres could upregulate the expression level of *Nanos3*, *Nobox* and *Lhx8*, and resume ovulation through regulation of the follicular microenvironment and stimulation of follicular development (Kim et al., 2018). Peng et al. also showed that the mRNA levels of these three genes in POF mice treated with BMSCs were significantly higher than those in the untreated group (Peng et al., 2018). Other follicular development-related genes include *Foxo3a* and *Foxo1*. Ding *et al.* found that UCMSCs on a collagen scaffold can activate primordial follicles *in vitro via* phosphorylation of FOXO3a and FOXO1, and transplantation of collagen/UCMSCs to the ovaries of POF patients can elevate estradiol concentrations, improve follicular development and increase the number of antral follicles (Ding et al., 2018).

Cytokines are critical regulators of folliculogenesis and ovulation. They contribute to creating an environment supporting follicle selection and growth, regulating cellular proliferation/differentiation, follicular survival/atresia and oocyte maturation (Field et al., 2014). The most important cytokines in POF treatments are TGF-β and IFN-γ. TGF-β superfamily members, including TGF-βs, AMH, activins, inhibins, BMPs and GDFs, impact several stages of follicular development (Trombly et al., 2009; Sanfins et al., 2018). According to Knight et al*.*, the positive TGF-β regulators of preantral follicle growth, include GDF-9 and BMP-15 of oocyte origin, activins of granulosal origin, BMP-4 and BMP-7 of thecal origin and TGF-β from theca and GCs; in contrast, AMH plays a negative role in preantral follicle development (Knight and Glister, 2006). However, the existing research conclusions are not consistent with each other. El-Derany et al*.* transplanted BMSCs to a γ-ray induced POF rats model and reported that BMSCs recovered the folliculogenesis process, upregulating *Foxo1*, *Gdf-9* and *Fst* gene expression accompanied by downregulating TGF-β (El-Derany et al., 2021), whereas Song et al*.* and Yin et al*.* found that MSC transplantation could increase the level of TGF-β and decrease the level of IFN-γ in POF models (Yin et al., 2018a; Song et al., 2018). Additionally, Ling et al*.* reported that amnion-derived mesenchymal stem cell transplantation can inhibit granulosa cell apoptosis and that the expression levels of AMH were significantly increased in the treatment group compared to the POF group (Ling et al., 2017). Zhang et al. and Mohamed et al*.* also found that after MSC transplantation, AMH expression in ovarian tissue was significantly higher than that in the POF group (Mohamed et al., 2018; Zhang et al., 2018).

Although the mechanism of MSCs on follicular development is not completely clear, most research agrees that MSC transplantation can promote the development and formation of primordial follicles, eggs and reduce the apoptosis of GCs. All of the involved genes and their correlated mechanisms are listed in Table 2.

**TABLE 2:** The effects of MSCs on follicular development.

Related gene/hormones/cytokines	Regulation of expression	Outcome of MSC treatment	References
Nanos3	Up	Reducing atretic follicle and increasing antral follicle and secondary follicle	Lai et al. (2014)
Nobox	Up		
Lhx8	Up		
Nanos3	Up	Stimulating follicular development and resuming ovulation	Kim et al. (2018)
Nobox	Up		
Lhx8	Up		
TGF-β	Up	Inhibiting follicular atresia and reducing the apoptosis of GCs in secondary follicles and cystic follicles	Knight and Glister (2006)
GDF-9	Up		
BMP-15	Up		
BMP-4	Up		
BMP-7	Up		
Foxo1	Up	Recovering the suppressed folliculogenesis process and promoting egg formation	El-Derany et al. (2021)
Gdf-9	Up		
Fst	Up		
TGF-β	Up	Promoting follicular growth	Song et al. (2018)
IFN-γ	Down	Inhibiting granulosa cell apoptosis	Zhao et al. (2018a)
AMH	Up	Increasing the number of follicles	Ling et al. (2017)
AMH	Up	Promoting follicular growth	Mohamed et al. (2018)
FOXO3a	Up	Promoting follicular development and maturation	Ding et al. (2018)
FOXO1	Up		

### MSCs and PGCs

Multiple studies have shown that MSCs can be induced and differentiate into PGCs. Fang et al*.* and Li et al. proved that CD61 could promote the differentiation of ADMSC into PGC-like cells through activation of the TGF-β pathway (Li et al., 2016; Fang et al., 2017). Wei et al*.* found that AMSC can be induced into PGC-like cells by BMP4 (Wei et al., 2016). Ge et al. found that when hfSDSCs were cultured in porcine follicle fluid, they may differentiate into both male and female germ cell-like cells (Ge et al., 2015). Park et al. proved that female mouse skin-derived stem cells could differentiate into ovarian-cell-like cells that are consistent with female germ, and ovarian follicle somatic cells. When ovarian cell-like cells are transplanted into ovariectomized mice, they restore the estrus cycle and serum estradiol levels (Park et al., 2014). Unfortunately, no *in vivo* research has reported whether MSC-differentiated germ cells can be fertilized and form embryos, and studies in this area are still lacking.

### MSCs Can Promote the Proliferation of GCs

OGCs are the most important stromal cells in the ovary, providing necessary nutrition for oocyte development and follicle maturation, participating in the regulation of gonadotropins that modulate oocyte development and maintaining the microenvironment of oocyte maturation through autocrine and paracrine mechanisms. GCs play an important role in all developmental stages of follicles. GCs abnormalities can lead to abnormal hormone secretion, follicular development disorders and even follicular atresia (Lai et al., 2014). Chemotherapy induces GC apoptosis by damaging DNA and activating apoptosis pathways, thus leading POF. Therefore, enhancing GC function and inhibiting GC apoptosis may effectively prevent POF (Bedoschi et al., 2016). Studies have shown that GCs and MSCs express some similar surface markers (Dzafic et al., 2014; Maleki et al., 2014). Transplanted MSCs are mainly located in the GC layer around follicles, suggesting that MSCs have a significant effect on follicle formation and ovulation (Manshadi et al., 2019).

MSCs can inhibit GC apoptosis and promote GC proliferation by releasing cytokines and hormones, upregulating proliferation-related genes and inhibiting apoptosis-related genes (He et al., 2018; Wang et al., 2020). Zhang et al. showed that PMSC transplantation could upregulate the expression of AMH and FSHR in GCs of POF mice, inhibit GC apoptosis and follicular atresia, and thus restore ovarian function (Zhang et al., 2018). Fu et al. also found that BMSC transplantation may reduce GC apoptosis and improve ovarian function by releasing VEGF, HGF, and IGF-1 and upregulating Bcl-2 expression (Fu et al., 2008). Ding et al. showed that coculturing of AMSCs and GCs might inhibit the apoptosis of GCs, and transplantation of AMSCs may improve ovarian function during natural aging by secreting HGF and EGF (Ding et al., 2018).

The underlying mechanism of MSC treatment of POF may be related to exosome-mediated microRNA modulation. Multiple studies have highlighted the potential therapeutic advantages of using exosomal miRNAs from MSCs for the treatment of various diseases and injuries, including POF. Yang *et al.* demonstrated that BMSC-derived exosomes prevent ovarian follicular atresia in POF rats via the delivery of miR-144-5p, which can decrease GC apoptosis by targeting the PTEN pathway (Yang et al., 2020). Xiao et al. found that miR-146a and miR-10a are rich in exosomes secreted by AFSCs. miR-146a can restore ovarian function by downregulating IRAK1 and TRAF632 expression and miR-10a can inhibit GC apoptosis and prevent follicular atresia by suppressing Bim and caspase-9 expression (Xiao et al., 2016). Sun et al. found that exosomes derived from UCMSCs may prevent and treat chemotherapy-induced OGC apoptosis *in vitro* by upregulating the expression level of Bcl-2 and downregulating the expression levels of caspase-3, Bax, cleaved caspase-3 and cleaved PARP (Sun et al., 2017). miR-21 is related to apoptosis. Studies have shown that MSC treatment suppresses the expression of PTEN and PDCD4 through upregulation of miR-21 and inhibiting the apoptosis of GCs (Fu et al., 2017). Sun et al. reported that miR-644-5p carried by MSC exosomes could regulate p53 signaling and inhibit GC apoptosis (Sun et al., 2019).

### MSCs Promote Angiogenesis

The establishment and remodeling of the ovarian vascular system is the basis of ovarian development and functional recovery. The follicles and corpus luteum can obtain nutritional support through ovarian blood vessels and transport hormones to target organs. Some researchers observed the distribution of BMSCs in ovaries by labeling specific markers of BMSCs and found that BMSCs were mainly distributed in the blood vessels of damaged ovaries (Liu et al., 2014), implying that BMSCs may play a role in ovarian blood vessels construction. Angiogenesis-related factors secreted by MSCs, such as VEGF, HGF, IGF and FGF, are increased in MSC-transplanted POF ovaries. VEGF and HGF have a synergistic effect and synergistically promote angiogenesis (Golocheikine et al., 2010). The combination of VEGF and HGF leads to an increased vascular diameter (Beilmann et al., 2004); VEGF promotes the length, area and branch point number of the induced vessels, while HGF contributes to vascular area growth (He et al., 2018). Wang et al. showed that MSCs could promote ovarian angiogenesis and reduce interstitial fibrosis by secreting VEGF, IGF-1, GCSF and HGF (Wang et al., 2017). Xia et al. demonstrated that MSC transplantation could enhance the expression levels of VEGF, FGF2 and angiogenin, significantly stimulate neovascularization and increase blood perfusion of the grafts in ovarian tissue (Xia et al., 2015). Zhang et al., Cho et al. and Park et al., also proved that MSC transplantation could repair damaged POF ovaries and promote ovarian development and function through angiogenesis (Zhang et al., 2017; Park et al., 2019; Cho et al., 2021).

Microvesicles are cell-derived membrane and cytoplasmic components. There are three subtypes of EVs: exosomes, microvesicles and apoptotic bodies. Exosomes and microvesicles can transfer mRNA, protein and lipids to target cells through surface-expressed ligands and surface receptors, thus affecting the phenotype and function of the target cells (Bidarimath et al., 2017). EVs have a therapeutic effect on female reproductive disorders, such as repairing injured endometrium, suppressing fibrosis of the endometrium, regulating immunity and anti-inflammation, and repressing the apoptosis of GCs in ovaries (Liao et al., 2021). Several studies have shown that MSC-derived microvesicles contain multiple pro-angiogenic proteins, such as VEGF and HGF (Merino-Gonzalez et al., 2016; Pakravan et al., 2017; Han et al., 2019; Shi et al., 2019). Yang et al. showed that UCMSC microvesicles transplantation in POI mice could induce angiogenesis by activating the PI3K/Akt signaling pathway and improve ovarian function (Yang Z. et al., 2019). Sun et al. found that miR-644-5p carried by BMSC-derived exosomes inhibited the apoptosis of ovarian GCs by targeting the p53 pathway (Sun et al., 2019); Zhang et al. also found that UCMSC-derived microvesicles can inhibit the apoptosis of GSs by downregulating the expression level of caspase-3 and upregulating the ratio of Bcl-2/Bax (Zhang J. et al., 2020).

### Anti-inflammatory and Immunomodulatory Effect of MSCs

POF is an autoimmune disease. Autoimmune dysfunction is one of the most important pathogeneses of POF, causing inflammatory reactions of the ovary, destroying the ultrastructure of follicular cells (such as zona pellucida damage, gap link rupture and mitochondrial swelling), causing apoptosis of ovarian cells, affecting the maturation and atresia of follicles and inducing a decline in ovarian function (Nelson, 2001; Luo et al., 2017). It has been reported that certain types of immune cells will expand in ovaries with POF and infiltrate into the ovarian tissue, indicating that they are involved in the inflammation associated with POF (van Kasteren et al., 2000; Chernyshov et al., 2001; La Marca et al., 2010; Wang et al., 2018).

The anti-inflammatory effect is a critical mechanism by which MSCs restore ovarian function. MSCs may inhibit the activation and proliferation of lymphocytes, inhibit the secretion of proinflammatory cytokines, inhibit the function of antigen-presenting cells, and convey regulatory messages to immune cells (Zhou et al., 2019). In contrast, since the ovaries of most POF patients are in inflammatory conditions, the presence of inflammatory cytokines is also crucial for the regulation of MSC immunological and regenerative functions. Beldi et al. proved that the tumor TNF-α-TNFR2 axis is necessary for MSCs to produce anti-inflammatory mediators (such as IL-10, TGFβ and NO) and sustain regenerative functions such as wound healing, complex tube formation and endothelial pro-angiogenic support (Beldi et al., 2020a; Beldi et al., 2020b). IFN-γ and MSCs have a synergistic effect on immunosuppression. They upregulate PGE2, HGF, IL-6 and TGF-1 in MSCs and induce MSCs to express IDO, promoting GC proliferation and increasing the number of follicles (Najar et al., 2016; Liang et al., 2018). Yin et al. showed that the level of proinflammatory IFN-γ increased and the level of anti-inflammatory TGF-β decreased in POF mice, whereas PMSC transplantation reversed this situation and improved ovarian function (Yin et al., 2018a). A study also showed that PMSCs increase the secretion of IL-10 by inhibiting NF-κB-mediated pro-inflammatory reactions and thus promote tissue repair (Wang et al., 2016).

Immune cells (Treg cells, NK cells, Th cells, etc.) are important pathogenic factors in several models of autoimmune diseases (Alvarez Arias et al., 2014; Gianchecchi et al., 2018; Zhang X.-M. et al., 2020; Sakaguchi et al., 2020). These results indicate that the interaction of MSCs and immune cells plays a critical role in regulating the inflammatory microenvironment of POF. Yin et al. showed that PMSC transplantation might restore the ovarian function of POF mice by balancing the ratios of Th17/Tc17 and Th17/Treg cells (Yin et al., 2018b). Lu et al. reported that the serum levels of IL-2 and IFN-*γ* secreted by Th1 cells increased, while IL-4 secreted by Th2 cells decreased in POF mice; however, after UMSC transplantation, the amounts of these cytokines were reversed (Lu et al., 2019). Yin et al. showed that UCMSC transplantation into POF mice upregulates the ratio of CD8^+^ Treg cells, which have a typical immunosuppressive function and can reduce immune rejection (Su et al., 2014; Yin et al., 2020).

### The Effect of MSCs on Oxidative Stress

Oxidative stress is a phenomenon of imbalance between the oxidative system and the antioxidant system caused by excessive ROS produced in cells. Reduction of ROS can protect the structure and function of ovarian mitochondria, increase the levels of antioxidant and antiapoptotic enzymes, and reduce apoptosis and oxidative damage of the ovary (He et al., 2018). Abumaree et al. indicated that cocultured PMSCs could reverse the destructive effect of OS on H_2_O_2_-treated endothelial cells and increase cell proliferation and migration (Abumaree et al., 2017). One study showed that ROS inhibit the expression and activity of TERT and induce POF (Jiang et al., 2018). MSCs can increase the production of antioxidant enzymes and inhibit ROS production through secretion of HGF, IL-6, IL-8, VEGF, BDNF and LIF and activation of the FOXO, NOQ1/MAPK, PI3K/Akt and Nrf2-ARE pathways (Amoroso et al., 2017). One study indicated that fMSCs upregulate MT1, JNK1, PCNA and AMPK levels and enhance antioxidant effects (Huang et al., 2019). Recently, it has been found that PMSC transplantation can reduce the levels of UCP-2, SOD1, reactive oxygen species and 8-hydroxydeoxyguanosine in POF rats, improving mitochondrial function *in vivo*, inhibiting oxidative stress and improving ovarian function (Zhang et al., 2016).

Using MSCs to treat POF is a sophisticated project. To better understand the mechanism by which MSCs improves ovarian functions, we summarized the cytokines and regulatory factors involved in the homing effect, follicular development, cell proliferation/apoptosis, angiogenesis, immunomodulation and oxidative stress processes, as shown in Table 3.

**TABLE 3:** Factors involved in the process of MSC treatment of POF.

Issues	Factors	Function	References
Follicular development	TGF-βs, AMH, BMPs, GDFs	Promoting follicular development.	Sanfins et al. (2018)
	TGF-β, GDF-9, BMP-15, BMP-4, BMP-7, AMH	Reducing GC apoptosis and promoting GC proliferation.	Knight and Glister (2006)
	TGF-β	Recovering the suppressed folliculogenesis process.	El-Derany et al. (2021)
	TGF-β, IFN-γ	Promoting follicular growth.	Song et al. (2018)
	AMH	Inhibiting GC apoptosis and promoting follicular growth.	Ling et al. (2017)
Primordial germ cells	CD61, TGF-β	Promoting MSCs different into PGC-like cells.	Fang et al. (2017)
	BMP4	Inducing MSC into PGC-like cells	Wei et al. (2016)
Proliferation of GC	AMH	Inhibiting GC apoptosis.	Zhang et al. (2018)
	VEGF, HGF, IGF-1, Bcl-2	Reducing GC apoptosis and improving ovarian function.	Fu et al. (2008)
	HGF, EGF	Reducing apoptosis of ovarian GC.	Ding et al. (2018)
	PARP	Inhibiting ovarian follicular atresia and reducing GC apoptosis.	Sun et al. (2017)
	Bcl-2, AMH, FSHR, caspase-3	Promoting GC proliferation and inhibiting GC apoptosis.	Wang et al. (2020)
Angiogenesis	VEGF, HGF	Promoting ovarian angiogenesis.	Golocheikine et al. (2010)
	VEGF, HGF	Increasing vascular diameter.	Beilmann et al. (2004)
	VEGF, IGF-1, GCSF, HGF	Promoting ovarian angiogenesis and reducing interstitial fibrosis.	Wang et al. (2017)
	VEGF, FGF2	Stimulating neovascularization and increasing blood perfusion of the grafts.	Xia et al. (2015)
Immunomodulatory effect	IL-2, IFN-γ, IL-4	Reducing GC apoptosis.	Lu et al. (2019)
Anti-inflammatory effect	PGE2, HGF, IL-6, TGF-1	Promoting GC proliferation.	Liang et al. (2018)
	IFN-γ, TGF-β	Improving ovarian function	Yin et al. (2018a)
Oxidative stress	HGF, IL-6, IL-8, VEGF, BDNF, LIF	Increasing the production of antioxidant enzymes and inhibiting ROS production.	Amoroso et al. (2017)

## Perspective

MSCs possess multiple differentiation potentials and homing and immunomodulatory functions. They can be used as seed cells to participate in the regeneration and reconstruction of tissues and organs in various diseases, such as rheumatoid arthritis, amyotrophic lateral sclerosis, systemic lupus erythematosus and other degenerative diseases (spinal cord injury, Parkinson’s disease, Alzheimer’s disease). At present, more than ten kinds of stem cell preparations have been used to treat graft-versus-host disease (Zhao et al., 2019), acute myocardial infarction (Cho et al., 2017), osteoarthritis (Matas et al., 2019), etc. The clinical application of MSCs has brought great hope to the treatment of POF infertility and the improvement of female reproductive health, and a large number of clinical studies are actively being carried out. However, with increasing age, the number and function of MSCs decrease accordingly. The senescence of MSCs may be related to telomere shortening, DNA damage, epigenetics and immunological characteristics (Trachana et al., 2017; Wagner, 2019). At present, senescence of MSC is still the bottleneck of stem cell tissue engineering and clinical applications. Therefore, how to deeply understand the molecular mechanism of MSC senescence and delay or prevent MSC senescence efficiently through reasonable gene manipulation or drug intervention, has crucial practical significance and important economic value.

## Conclusion

MSCs derived from different sources have similar curative effects in the treatment of POF through multiple mechanisms. MSCs have attractive clinical transformation and application prospects in the restoration of reproductive function in POF patients, even in older women with POF. Therefore, understanding the molecular mechanism of POF is still a key scientific problem for comprehensively and deeply evaluating the safety and effectiveness of MSC transplantation, especially the long-term impact on parents and offspring.

## Glossary

ADMSC: adipose-derived stem cells
AFMSC: amniotic fluid mesenchymal stem cells
akt: protein kinase B
AMH: anti-mullerian hormone
AMPK: adenosine 5‘-monophosphate (AMP)-activated protein kinase
AMSC: amniotic mesenchymal stem cells
ARE: antioxidant response element
AZPAb: anti-Zona pellucida antibody
Bax: Bcl-2 associated X protein
Bcl-2: B-cell lymphoma-2
b-FGF: basic fibroblast growth factor
BDNF: brain-derived neurotrophic factor
Bim: Bcl-2 interacting mediator of cell death
BMP: bone morphogenetic protein
BMSC: bone marrow stem cells
CD: cluster of differentiation
EGF: epidermal growth factor: epidermal growth factor
E2: estrogen
EGF: epidermal growth factor: epidermal growth factor
FGF: fibroblast growth factor
FOXO: forkhead box O
FSH: follicle stimulating hormone
FSHR: follicle stimulating hormone receptor
fst: homo sapiens follistatin
GC: ovarian granulosa cells: granulosa cells
GC: ovarian granulosa cells: granulosa cells
GCSF: granulocyte colony stimulating factor
Gdf: growth differentiation factor
HGF: hepatocyte growth factor
HOVEC: human ovarian endothelial cell
HRT: hormone replacement therapy
IDO: indoleamine 2,3-dioxygenase
IGF-1: insulin-like growth factors-1
IFN-γ: interferon γ
IRAK1: interleukin 1 receptor associated Kinase 1
IL: interleukin-10
JNK1: jun n-terminal kinase1
LH: luteinizing hormone
LIF: interleukin 6 family cytokine
MAPK: mitogen-activated protein kinase
MCP: monocyte chemotactic protein
Mensc: menstrual-derived stem cell
MSC: mesenchymal stem cells
MVs: microvesicles
MT1: melatonine receptor1
Nanos3: nanos C2HC-type Zinc finger 3
NF-κB: nuclear factor kappa-light-chain-enhancer of activated B cells
NK: natural killer cell
Nobox: NOBOX oogenesis homeobox
NO: nitric oxide
NOQ1: NAD(P)H quinone dehydrogenase 1
Nrf2: NF-E2-related factor 2
OGCs: ovarian granulosa cells
PARP: poly ADP-ribose polymerase
PCNA: proliferating cell nuclear antigen
PDCD4: programmed cell death 4
PDMSC: placenta-derived mesenchymal stem cells
PGF: placental growth factor
PGE2: prostaglandin E2
PGC: primordial germ cell
PMSC: placenta-derived mesenchymal stem cell
POF: premature ovarian failure
PTEN: phosphatase and tensin homolog
PI3K: phosphatidylinositol-3-kinase
ROS: reactive oxygen species
SC: stem cell
SOD1: superoxide dismutase 1
TERT: telomerase reverse transcriptase
TGF: transforming growth factor
th: helper T cell
TNF-α: tumor necrosis factor-α
TRAF632: receptor associated factor 632
UCMSC: umbilical cord mesenchymal stem cells
UCP-2: uncoupling protein-2
VEGF: vascular endothelial growth factor
VEGFR: vascular endothelial growth factor
